# Bibliometric analysis of biologic treatments for chronic rhinosinusitis

**DOI:** 10.3389/fmed.2025.1623940

**Published:** 2025-08-11

**Authors:** Yue Huang, Yulan Chen, Qihong Li, Wen Sui, Zhuo Pan, Hao Yang, Zeyi Lv, Xi Chen, Man Yin, Yu Li, Xinrong Li

**Affiliations:** Hospital of Chengdu University of Traditional Chinese Medicine, Chengdu, China

**Keywords:** chronic rhinosinusitis, biologic treatments, bibliometric, visualized analysis, emerging trends

## Abstract

**Background:**

Chronic rhinosinusitis (CRS) significantly impacts patients’ quality of life. The use of biologic therapies in CRS management has gained traction in clinical practice. However, no bibliometric analysis has been conducted in this area thus far. This study aims to provide a comprehensive overview of the knowledge framework and research trends regarding biologic treatments for CRS.

**Methods:**

A bibliometric analysis was performed on 888 publications related to biologic treatments for CRS, published between 2011 and 2024. Literature was retrieved from the Web of Science (WoS), and data visualization and trend analysis were conducted using VOSviewer, CiteSpace, and Bibliometrix software tools.

**Results:**

Research on biologic therapies for CRS peaked in the past 6 years. Key contributors include Claus Bachert, the United States, and the University of Ghent. The most cited article is “Efficacy and safety of dupilumab in patients with severe chronic rhinosinusitis with nasal polyps (LIBERTY NP SINUS-24 and LIBERTY NP SINUS-52): results from two multicenter, randomized, double-blind, placebo-controlled, parallel-group phase 3 trials.” The five most explosive keywords are: expression (5.03), placebo-controlled trial (3.68), anti-IgE (3.35), anti-IgE antibody (3.22), and phenotypes (4.55). Current research on biologic treatments for CRS predominantly focuses on clinical applications.

**Conclusion:**

This study offers a bibliometric visualization of the literature on biologic treatments for CRS, highlighting key developments and emerging research trends in the field. It provides valuable references for scholars and outlines future research directions to further advance the field.

## 1 Introduction

Chronic rhinosinusitis (CRS) is a prevalent condition characterized by persistent inflammation of the nasal and paranasal sinus mucosa, affecting approximately 5–12% of the global population (1). It significantly diminishes patients’ quality of life and exerts a substantial socioeconomic impact. Traditional treatments typically involve nasal and short-term oral corticosteroids (OCS). For patients unresponsive to these therapies, endoscopic sinus surgery offers an alternative (2). However, 30–60% of patients with refractory CRS continue to experience recurrent symptoms and inadequate therapeutic outcomes, particularly those with type 2 inflammatory phenotypes and nasal polyps. In recent years, biologic therapies targeting specific inflammatory pathways, such as IL-4, IL-5, IL-13, and IgE, have revolutionized the treatment of Th2-related diseases (3, 4), including asthma and atopic dermatitis. These developments provide new opportunities for the targeted treatment of CRS. The number of clinical trials and observational studies investigating biologic agents for CRS (e.g., omalizumab, dupilumab) has increased significantly.

Biologic agents have transformed the treatment landscape for CRS, driving rapid advancements in research within this field. However, no comprehensive bibliometric analysis has yet been conducted to systematically assess the knowledge structure, research hotspots, and evolving trends. Existing literature is dispersed across multidisciplinary journals in immunology, otolaryngology, and pharmacy, making it difficult for traditional reviews to objectively quantify the integration of these diverse fields. Narrative reviews are often constrained by the author’s subjective selection and interpretation, which can lead to overlooking pivotal studies or underestimating emerging trends. Systematic reviews or meta-analyses typically focus on specific clinical questions, failing to address broader trends within the field. Therefore, this study utilizes bibliometric methods to analyze the current landscape, research evolution, and future directions of biologic treatments for CRS, identifying key contributors (countries, institutions, authors) and foundational knowledge. The purpose of this study is to identify major contributing countries, regions, institutions, and core authors; map the distribution of global research forces; reveal international cooperation networks; and accurately quantify publication volume, growth trends, research activity levels, and developmental stages in this field. And it aims to bridge existing gaps and provide a scientific basis for advancing basic research, clinical practice, and resource allocation, while also serving as a reference for optimizing the clinical application and guidelines surrounding biologic treatments. A systematic survey and perspective of the existing literature on biologic treatments for CRS provides researchers, policymakers, funders, and other stakeholders with essential macro-level insights to advance the field. This comprehensive understanding is difficult to achieve systematically, objectively, and quantitatively through individual studies or narrative reviews.

## 2 Materials and methods

### 2.1 Data collection

**Figure 1:**
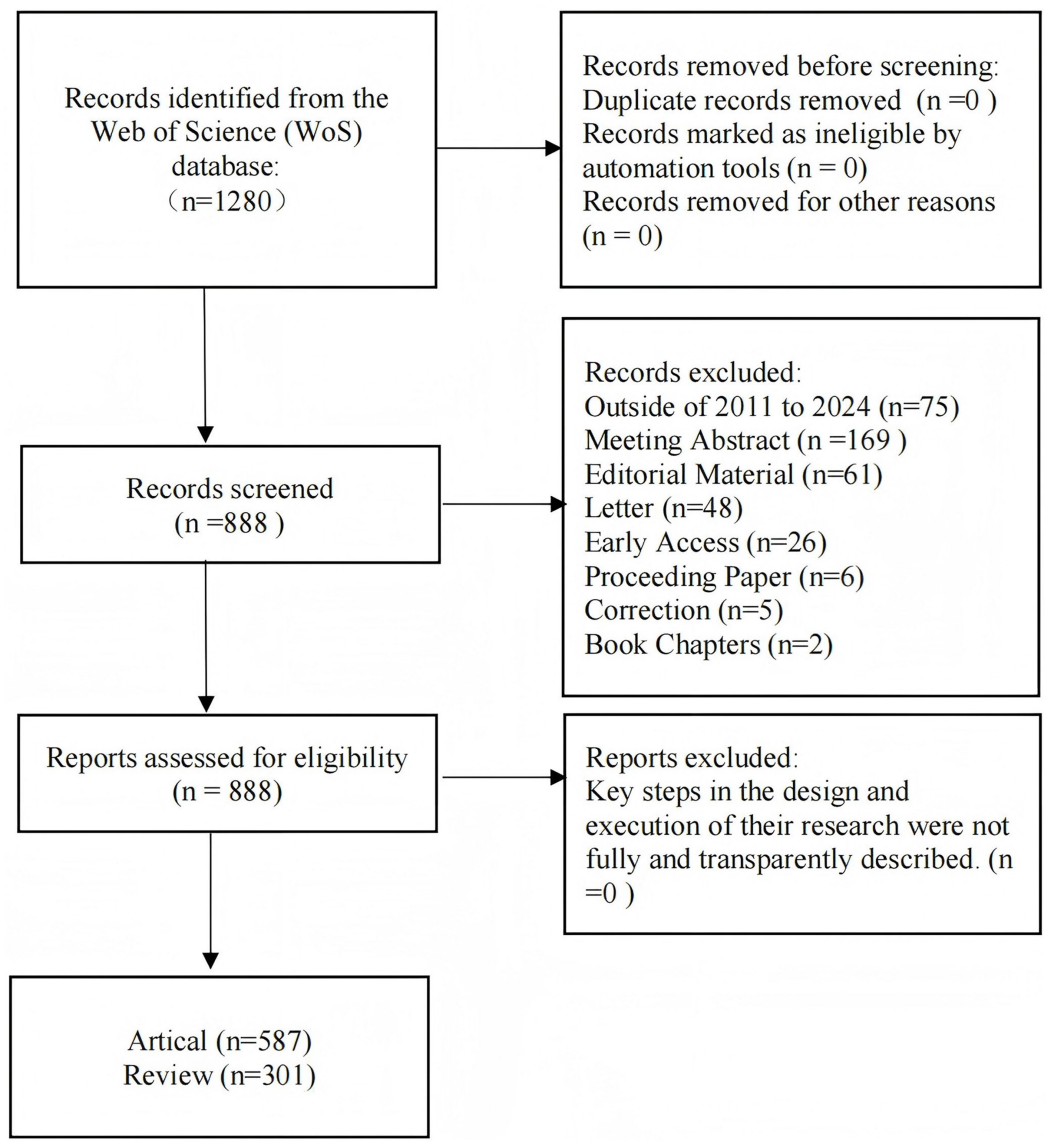
Publication screening flowchart.

The bibliometric analysis was conducted using the Web of Science (WoS) database. The search query was configured as [(TS = chronic rhinosinusitis) OR (TS = nasal polyps)] AND [(TS = biologics) OR (TS = dupilumab) OR (TS = omalizumab) OR (TS = mepolizumab) OR (TS = tezepelumab)]. A total of 1,280 publications were obtained. No duplication and no records marked as unqualified by automated tools. Publications excluding meeting abstract (*n* = 169), editorial material (*n* = 61), letter (*n* = 48), early access (*n* = 26), correction (n = 6), procceding paper (*n* = 5), book chapters (*n* = 2) and outside 2011 to 2024 (*n* = 75) were excluded. The selected publication types were articles (*n* = 587) and reviews (*n* = 301). The study results were saved in “plain text” format and exported as “full record.” The data retrieval and collection process is outlined in Figure 1. Two independent reviewers (YH, YLC) performed the study selection, data extraction, and quality assessment.

### 2.2 Data analysis

Bibliometric networks were constructed and visualized using VOSviewer software (version 1.6.18), which provides text mining capabilities to create and display co-occurrence networks of key terms extracted from scientific literature (5). CiteSpace (version 6.1.3) was used to visualize the progressive development of the knowledge domain, with a focus on identifying significant milestones, particularly intellectual and pivotal turning points in the field (6). The R package ‘bibliometrix’ (version 4.0.1), an open-source tool for quantitative research in scientometrics and bibliometrics, was employed to conduct comprehensive bibliometric analyses, incorporating various bibliometric methods (7).

## 3 Results

### 3.1 Global trend in publication outputs

**Figure 2:**
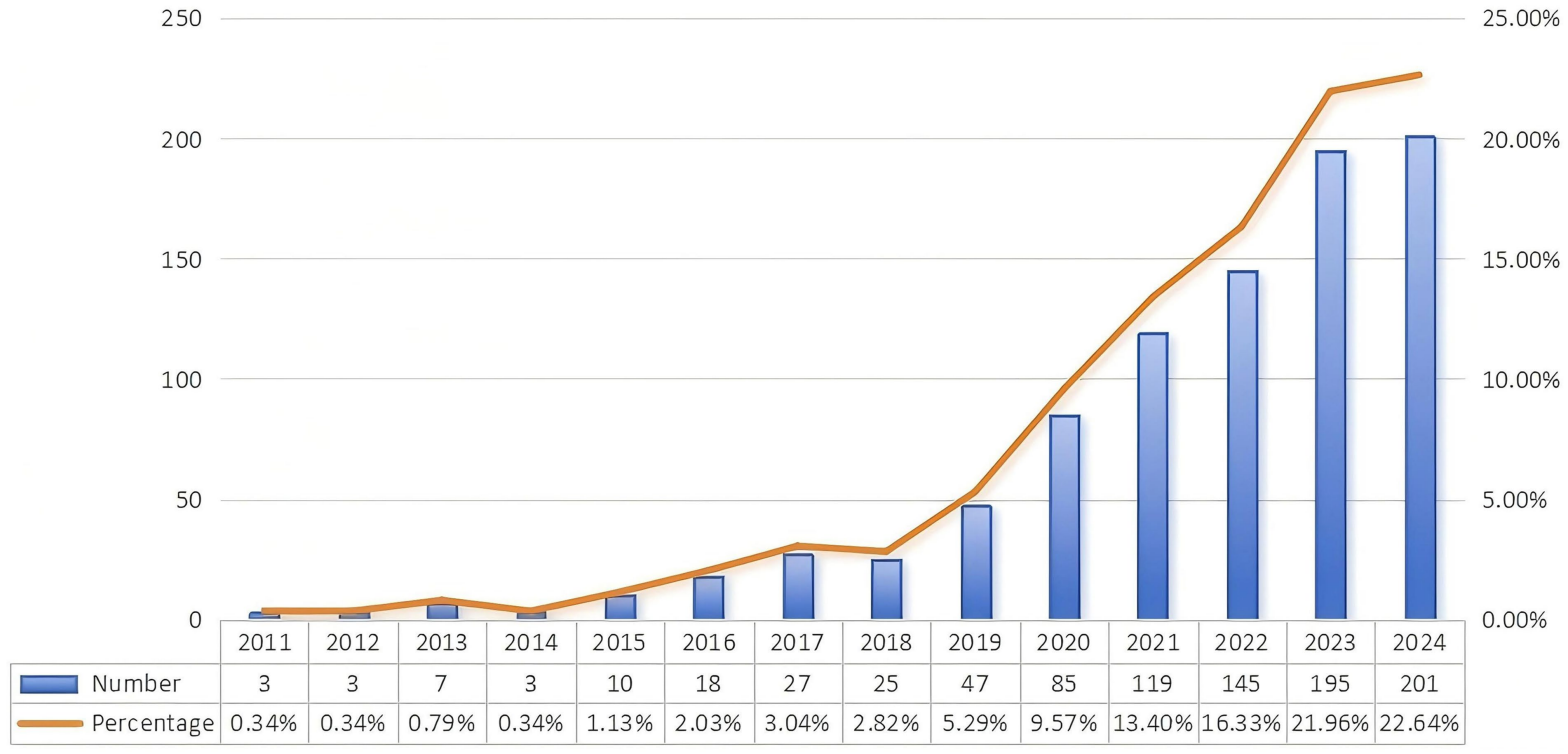
Global trend in publications. It shows the significant changes in the number of publications in biologic treatments for CRS and its proportion in the total amount from 2011 to 2024, showing an overall rapid growth trend, especially after 2019, when the growth rate accelerated significantly.

Figure 2 illustrates the global trend in publications from 2011 to 2024. Research on biologic treatments for CRS has experienced rapid growth in the past 6 years, accounting for 90% of all publications in this field. The number of global articles increased annually from 3 in 2011 to 201 in 2024. Initially, this area of research was underdeveloped, but since 2019, there has been a significant surge in publications, reflecting the growing interest and attention to biologic treatments for CRS. The consistent high volume of publications indicates that this remains a prominent research topic. The rapid advances in biologic treatments for CRS directly reflect the medical community’s commitment to overcoming disease heterogeneity, identifying targeted pathways and biomarkers, and advancing precision medicine by matching biologics to individual patient profiles. This research acceleration is driven by a paradigm shift: from empirical, one-size-fits-all approaches toward personalized, biomarker-guided precision therapies.

### 3.2 National and institutional analyses

**Figure 3:**
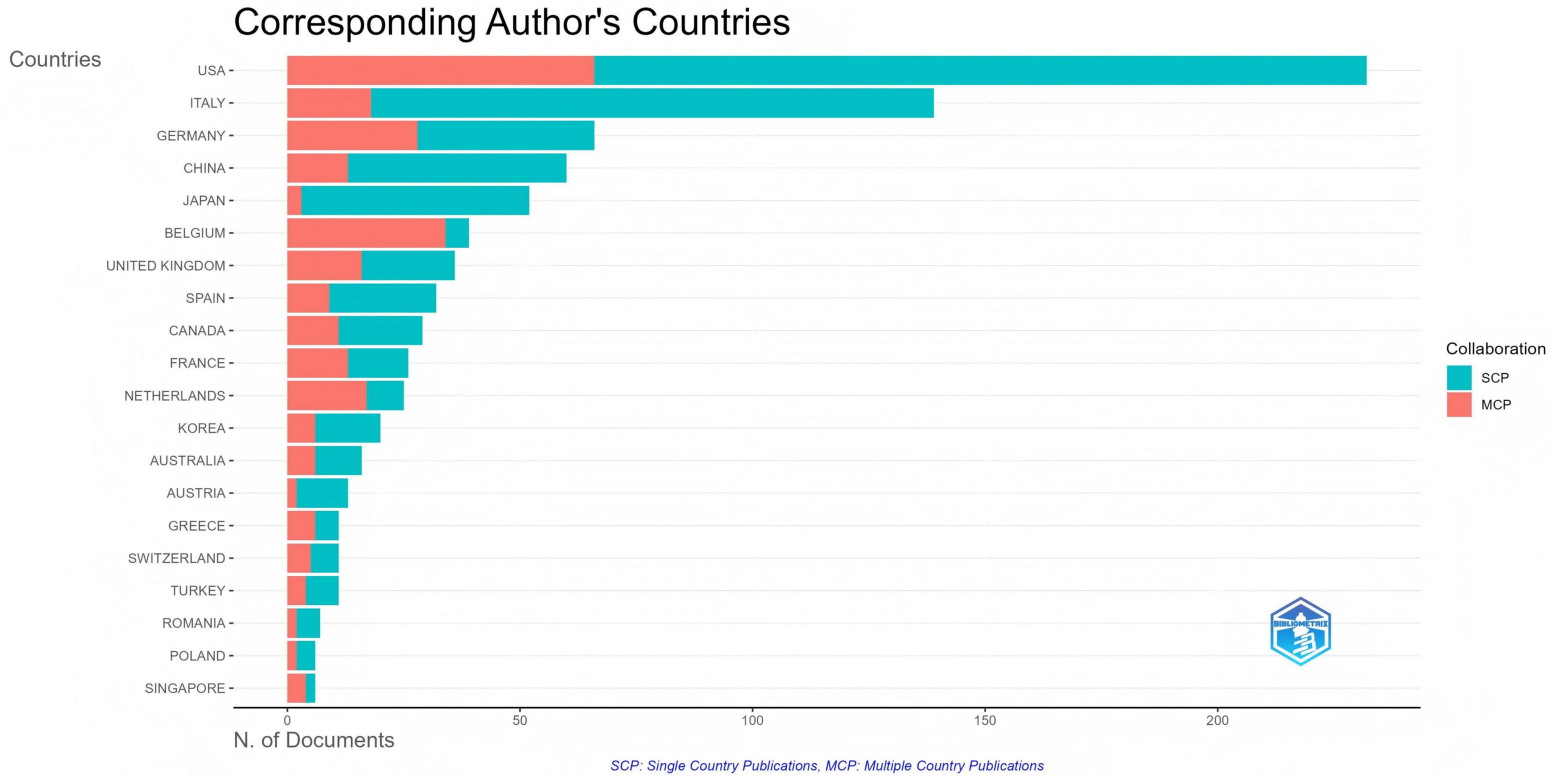
Top 20 countries of corresponding authors in research on biologic treatments for CRS. Countries are ranked from highest to lowest in terms of the total number of publications (the longer the bar, the more publications). Blue represents the number of publications by a single country, and red represents the number of publications by multiple countries.

A total of 58 countries and 1,623 institutions have contributed to research on biologic treatments for CRS, with the top 10 most productive countries listed in Table 1. The United States led the field with the highest number of publications (*n* = 232), followed by Italy (*n* = 139) and Germany (*n* = 66). Together, the United States and Italy accounted for over 40% of the total publications. The United States also had the highest total citations, highlighting its leading role in this research area. Multinational publications (MCPs), which represent collaborative contributions from multiple countries, were analyzed to assess international cooperation. Germany and Belgium stood out with a high percentage of MCPs (Figure 3), demonstrating their substantial role in international collaboration. While Belgium published fewer articles, its research exchange with other nations remained robust.

**Table 1:** Top 10 productive countries with publications on research of biologic treatments for CRS.

Country	Articles	Articles %	SCP	MCP	Country	TC	Average article citations
United States	232	26.1	166	66	United States	7,574	32.60
Italy	139	15.7	121	18	Belgium	4,758	122.00
Germany	66	7.4	38	28	Italy	2,298	16.50
China	60	6.8	47	13	Netherlands	1,286	51.40
Japan	52	5.9	49	3	China	1,241	20.70
Belgium	39	4.4	5	34	Japan	1,071	20.60
United Kingdom	36	4.1	20	16	United Kingdom	878	24.40
Spain	32	3.6	23	9	Spain	678	21.20
Canada	29	3.3	18	11	Germany	573	8.70
France	26	2.9	13	13	Canada	502	17.30

SCP, single country publication; MCP, multiple country publication; TC, total citation.

**Figure 4:**
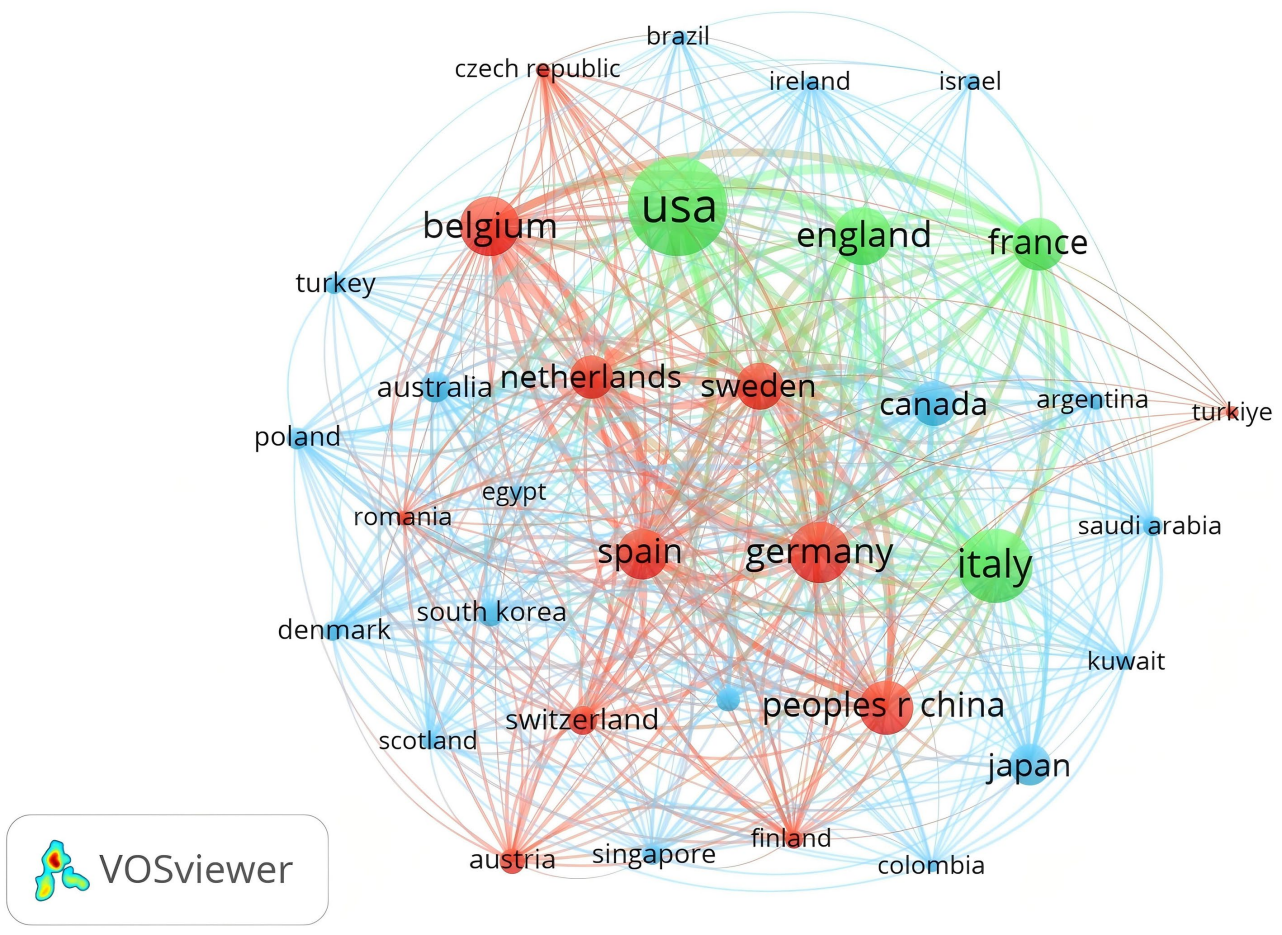
Analysis of countries related to biologic treatments for CRS. Visual map of national/regional citation networks. The size of each circle/node represents the number of publications, with line thickness indicating the strength of connections between circles/nodes. Clusters of related objects are color-coded, with each circle/node representing a separate country or region.

A minimum threshold of 5 articles was applied to filter the 37 countries meeting this criterion. Figure 4 illustrates the close collaboration between these nations, illustrating an extensive research network with the United States and European countries as key hubs in the global scientific community.

Among the top 10 institutions by publication count, Ghent University stands out as the most productive, with 64 publications and 5,529 total citations. It is followed by Harvard Medical School (*n* = 57) and Sanofi (*n* = 55; Table 2). These institutions not only contribute significantly in terms of publication volume but also in terms of impact. For example, while Karolinska Institutet ranks sixth in publication count (*n* = 41), it ranks second in total citations (TC = 4,038), reflecting the high quality and influence of its research.

**Table 2:** Top 10 central institutions studying on research of biologic treatments for CRS.

Rank	Institutions	NP	TC	Countries
1	University of Ghent	64	5,529	Belgium
2	Harvard Medical School	57	2,447	United States
3	Sanofi	55	3,504	French
4	Regeneron Pharmaceuticals, Inc	49	2,596	United States
5	Northwestern University	47	1796	United States
6	Karolinska Institutet	41	4,038	Sweden
7	University of Barcelona	41	3,089	Spain
8	Sun Yat-sen University	37	1,364	China
9	University of Amsterdam	34	1,635	Netherlands
10	Eastern Virginia Medical School	33	3,362	United States

NP, number of publications; TC, total citation.

**Figure 5:**
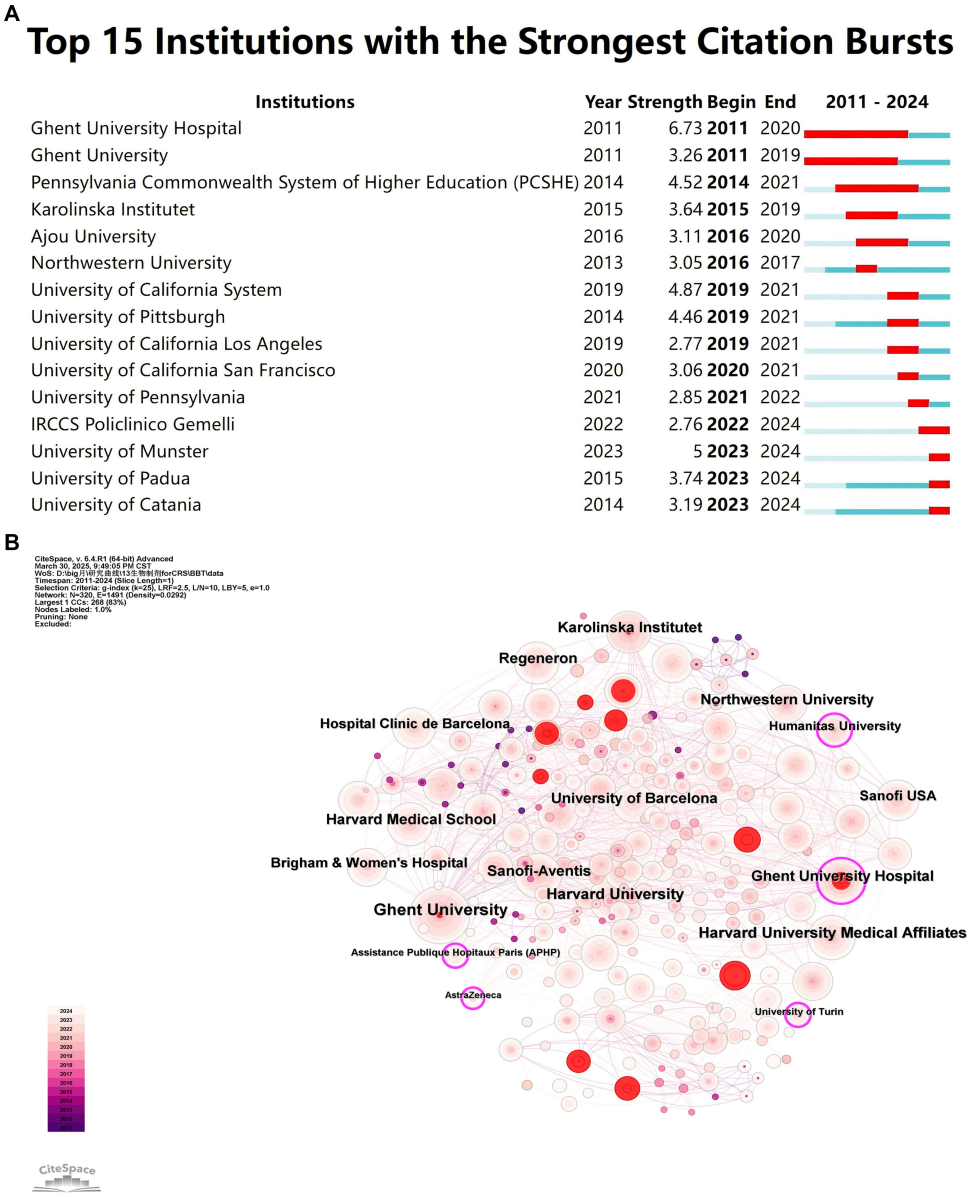
**(A)** Co-author–institution collaboration visualization in biologic treatments for CRS. It refers to the phenomenon that the citation frequency of academic achievements of an institution increases abnormally in a specific period of time, reflecting the sudden influence of its research. A red bar indicates high citation counts for that year. **(B)** Co-occurrence map of research institutions. The node size reflects co-occurrence frequencies, while links represent co-occurrence relationships between institutions. Nodes with purple rounded corners signify high betweenness centrality (≥ 0.1).

Figure 5A highlights the top 15 institutions with citation outbreaks. Notable institutions, such as IRCCS Policlinico of Pennsylvania, University of Münster, University of Padua, and University of Catania, have recently experienced citation surges, indicating their growing impact in biologic treatments for CRS. Ghent University Hospital and Ghent University experienced early citation outbreaks that lasted nearly a decade, underscoring Belgium’s solid foundation and leading position in this field. Figure 5B presents a co-occurrence network where node size represents the frequency of co-occurrence, and links indicate the relationships between co-occurring institutions. Nodes with purple rounded corners represent institutions with high mediator centrality (≥0.1). Institutions such as Ghent University Hospital, with high centrality, play a critical role in linking diverse research communities.

### 3.3 Analysis of journals

**Figure 6:**
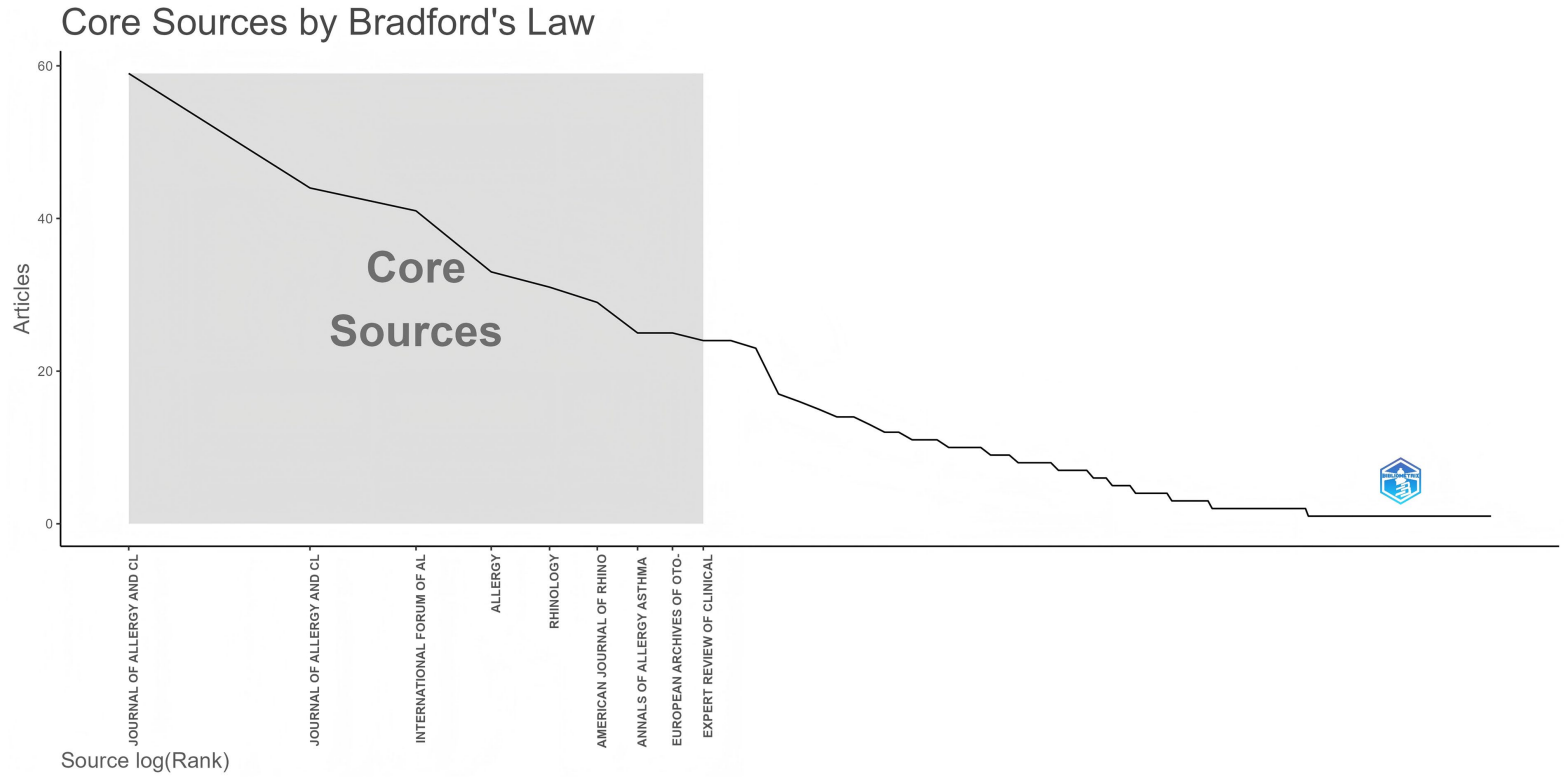
Analysis of academic journals related to biologic treatments for CRS, based on Bradford’s law. Bradford’s Law reveals the concentration of literature—a small number of core journals contribute most important papers. Top journals are the absolute core of the field, carrying the highest density of important literature.

To identify active and influential journals, a visual analysis of the journals publishing research on biologic treatments for CRS was conducted. A total of 888 publications across 183 academic journals were identified. As shown in Table 3 and Figure 6, the Journal of Allergy and Clinical Immunology led with the most publications (NP: 59), followed by the Journal of Allergy and Clinical Immunology-In Practice (NP: 44). Among the top 10 journals, Allergy boasts the highest impact factor (9.8), underscoring its significant influence in the field of biologic treatments for CRS.

**Table 3:** Top 10 influential academic journals with publications concerning biologic treatments for CRS.

Source	h_index	TC	NP	IF	JCR
JOURNAL OF ALLERGY AND CLINICAL IMMUNOLOGY	27	4,170	44	8.9	Q1
JOURNAL OF ALLERGY AND CLINICAL IMMUNOLOGY-IN PRACTICE	25	2,274	59	5.3	Q1
ALLERGY	22	1705	33	9.8	Q1
INTERNATIONAL FORUM OF ALLERGY & RHINOLOGY	15	681	41	3.9	Q1
RHINOLOGY	15	764	31	5.2	Q1
ANNALS OF ALLERGY ASTHMA & IMMUNOLOGY	12	485	25	3.6	Q1
AMERICAN JOURNAL OF RHINOLOGY & ALLERGY	11	514	29	2.2	Q1
EXPERT REVIEW OF CLINICAL IMMUNOLOGY	11	303	24	3.2	Q2
ALLERGY AND ASTHMA PROCEEDINGS	10	185	12	1.9	Q2
CLINICAL AND EXPERIMENTAL ALLERGY	10	678	11	3.8	Q1

h_index, Hirsch index; NP, number of publications; TC, total citation; IF, impact factor.

### 3.4 Author contributions and co-occurrence

**Figure 7:**
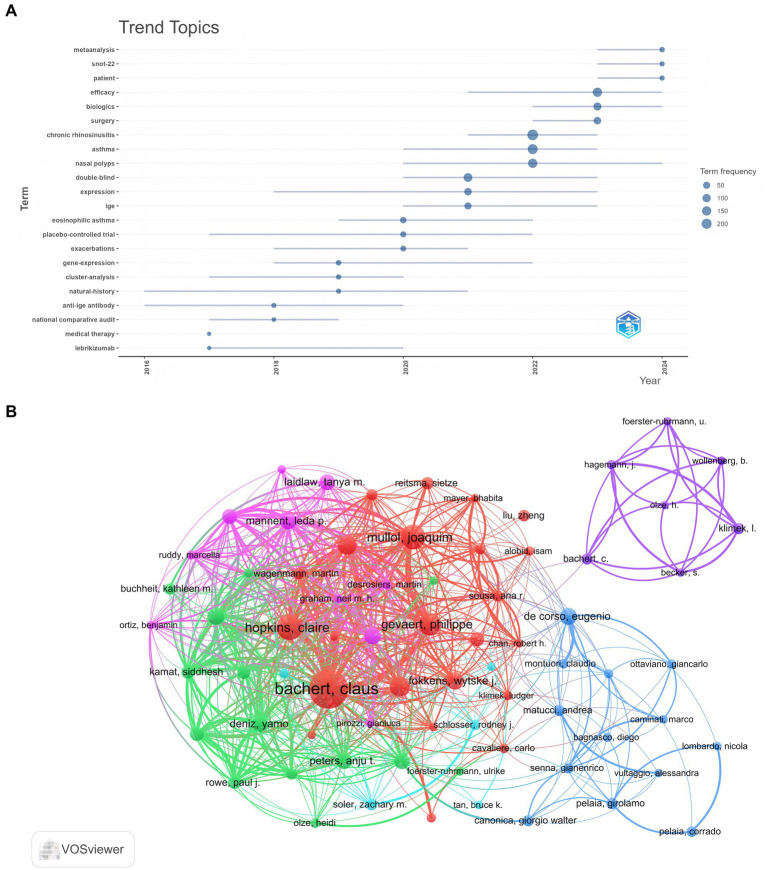
**(A)** Trend topic analysis of biologic treatments for CRS, covering themes from 2011 to 2024. The timeline illustrates the temporal progression of key research themes, with fluctuations in their prominence. Larger nodes indicate increased frequency and significance of these themes. **(B)** Analysis of authors in biologic treatments for CRS. The varied colored nodes (73 in total) represent authors in different clusters. The node size reflects co-occurrence frequencies, while the linkages represent co-occurrence relationships between authors.

A total of 3,736 authors contributed to this research, with the top 10 most relevant authors listed in Table 4. Bachert Claus emerged as the leading author, with 37 articles and 7,287 citations, followed by Gevaert Philippe, who published 24 articles and garnered 3,973 citations. Figure 7A illustrates the temporal distribution of author productivity, where circle size indicates the number of publications, and color reflects total citations per year. Notably, Han, Joseph K achieved 136 total citations in 2019, despite publishing only one article that year. Figure 7B presents the collaborative network among 73 authors involved in international research on biologic therapies for CRS. Claus Bachert demonstrated the highest level of international collaboration, with a total link strength of 350, reflecting his extensive and robust research network. Claire Hopkins followed closely with a link strength of 200, indicating significant collaborative efforts. Joaquim Mullol and Philippe Gevaert also exhibited substantial collaboration, with link strengths of 185 and 173, respectively.

**Table 4:** Top 10 most relevant authors and their production.

Author	h_index	TC	NP
BACHERT CLAUS	37	7,287	77
GEVAERT PHILIPPE	24	3,973	37
HOPKINS CLAIRE	21	3,166	44
MULLOL JOAQUIM	21	3,731	41
MANNENT LEDA P.	18	2,191	23
LAIDLAW TANYA M.	17	2,117	22
AMIN NIKHIL	16	1958	20
HAN JOSEPH K.	16	2,383	29
HEFFLER ENRICO	16	1,250	27
HELLINGS PETER W.	16	2,107	25

NP, number of publications; TC, total citation.

### 3.5 Citation analyses

Among the top 8 cited papers (Table 5), the article by Claus Bachert holds the highest number of citations, highlighting his central role in the field. Four of these papers were published in the Journal of Allergy and Clinical Immunology, with Philippe Gevaert contributing to three of them. These studies provide compelling evidence for the significant efficacy of pathway-specific biologics in targeted patient subgroups, advancing the development and implementation of personalized precision therapy strategies in CRS management.

**Table 5:** Top 8 most globally cited documents concerning biologic treatments for CRS.

Rank	Title	First author	Journal	Year	TC
1	Efficacy and safety of dupilumab in patients with severe chronic rhinosinusitis with nasal polyps (LIBERTY NP SINUS-24 and LIBERTY NP SINUS-52): results from two multicenter, randomized, double-blind, placebo-controlled, parallel-group phase 3 trials	BACHERT C	LANCET	2019	956
2	Effect of Subcutaneous Dupilumab on Nasal Polyp Burden in Patients With Chronic Sinusitis and Nasal Polyposis: A Randomized Clinical Trial	BACHERT C	JAMA-J AM MED ASSOC	2016	607
3	Reslizumab for poorly controlled, eosinophilic asthma: a randomized, placebo-controlled study	CASTRO M	AM J RESP CRIT CARE	2011	560
4	Omalizumab is effective in allergic and nonallergic patients with nasal polyps and asthma	GEVAERT P	J ALLERGY CLIN IMMUN	2013	545
5	Efficacy and safety of omalizumab in nasal polyposis: 2 randomized phase 3 trials	GEVAERT P	J ALLERGY CLIN IMMUN	2020	457
6	Mepolizumab, a humanized anti-IL-5 mAb, as a treatment option for severe nasal polyposis	GEVAERT P	J ALLERGY CLIN IMMUN	2011	437
7	Reduced need for surgery in severe nasal polyposis with mepolizumab: Randomized trial	BACHERT C	J ALLERGY CLIN IMMUN	2017	380
8	IL-1β, IL-4 and IL-12 control the fate of group 2 innate lymphoid cells in human airway inflammation in the lungs	BAL SM	NAT IMMUNOL	2016	371

### 3.6 Analysis of keywords and hotspots

**Figure 8:**
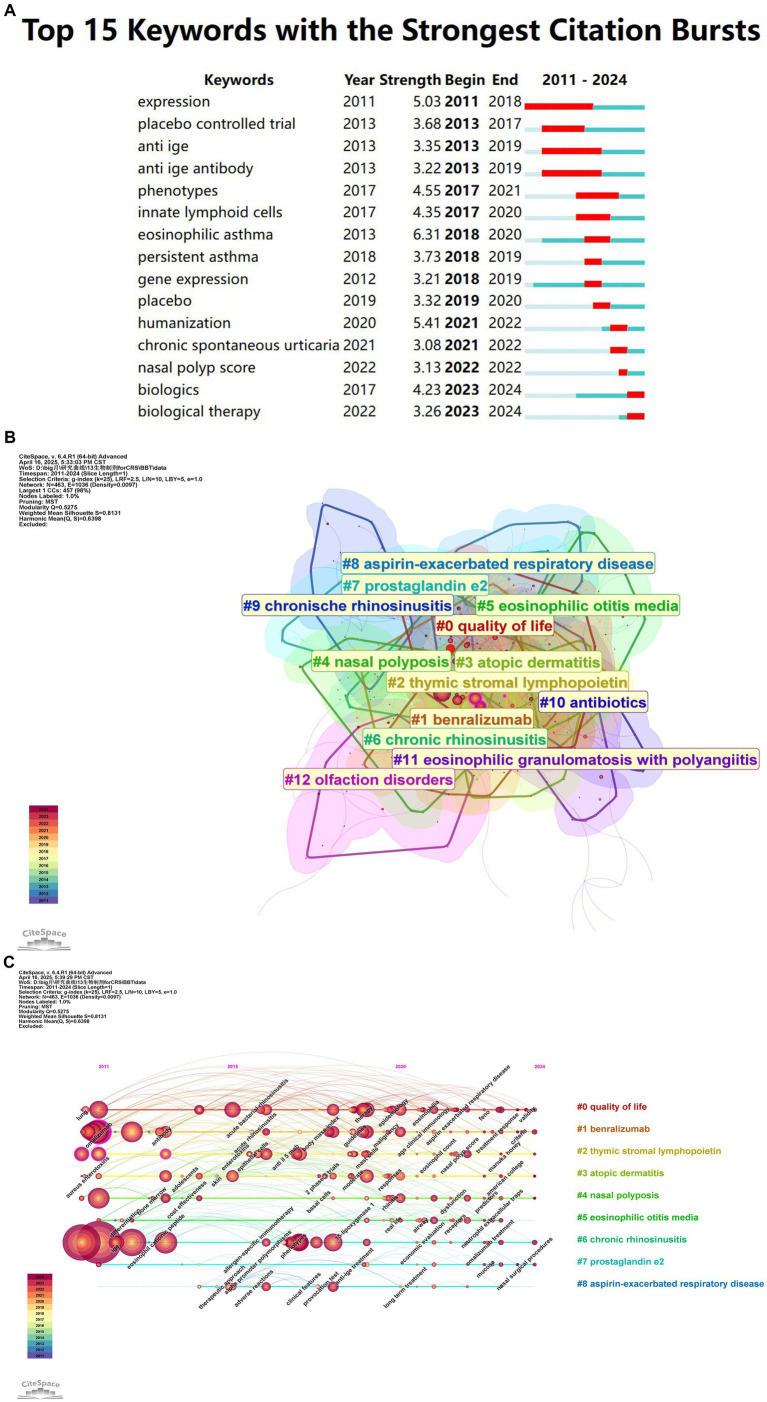
**(A)** Top 15 keywords with the strongest citation bursts. It refers to the phenomenon that the citation frequency of keyword increases abnormally in a specific period of time, reflecting the sudden influence of its research. A red bar indicates high citation frequency during that year. **(B)** Keyword cluster analysis co-occurrence map based on CiteSpace. Clustering of the co-occurrence keywords network, where smaller numbers indicate larger clusters. Larger circles represent keywords with more references during the corresponding period. **(C)** Timeline view of the keyword co-occurrence map. The timeline visualizes the temporal evolution of key research topics, with the salience of each keyword changing over time. Larger and more concentrated nodes represent higher frequency and importance, and the keywords are organized into clusters on the right-hand side of the figure.

Burst word detection algorithms reveal emerging research trends by analyzing the rate of increase in keyword occurrences. The red line represents the burst period, while the blue line indicates the time intervals. Popular research frontiers are examined based on the timing and duration of keyword bursts. Figure 8A presents the top 15 keywords with the most significant citation bursts. The five most intense keywords, in terms of explosive growth, are: expression (5.03), placebo-controlled trial (3.68), anti-IgE (3.35), anti-IgE antibody (3.22), and phenotypes (4.55). Notably, “biologics” and “biological therapy” continue to exhibit persistent bursts, suggesting that targeted therapy will continue to dominate the future of CRS research.

To better capture the research frontiers of biologic therapies for CRS, keyword cluster analysis was performed using a spectral clustering algorithm. The Log-Likelihood Ratio (LLR) algorithm was applied to extract keywords from cited articles and annotate the clusters. Twelve clusters were identified, with a modularity Q value of 0.5275 and a silhouette S value of 0.8131 (Figure 8B), confirming the robustness and reliability of the findings. The high overlap among the clustered color blocks indicates strong interrelationships and interactions between the different research areas. The keyword timeline view highlights the evolving research hotspots and their development over time.

Figure 8C provides a clear depiction of the temporal evolution and development of biologic therapies for CRS, illustrating the progression of research in this field. Research initially focused on foundational disease classification, followed by identifying key therapeutic targets and developing targeted drugs. Subsequent rigorous clinical trials validated therapeutic efficacy in specific populations, while ongoing exploration of molecular mechanisms uncovered novel biomarkers and refined disease subtypes. This progression exemplifies how scientific inquiry advances toward deeper understanding and greater precision.

## 4 Discussion

This study identified 888 publications on biologic therapies for CRS from 2011 to 2024, sourced from the WoS. A dramatic increase in publications occurred after 2019, with the United States leading in both publication volume and citation count. This surge was largely driven by the success of dupilumab in two Phase III clinical trials (LIBERTY NP SINUS-24 and SINUS-52) in 2019 (8). The results, published in the New England Journal of Medicine (NEJM) in 2019, marked a significant milestone and catalyzed the subsequent expansion of research. Based on these trials, the US FDA approved dupilumab for the treatment of CRS with nasal polyps (CRSwNP) in adults in June 2019, making it the first biologic approved for this indication, followed by approval from the European Medicines Agency (EMA). This milestone greatly enhanced academic and clinical interest in biologics.

**Figure 9:**
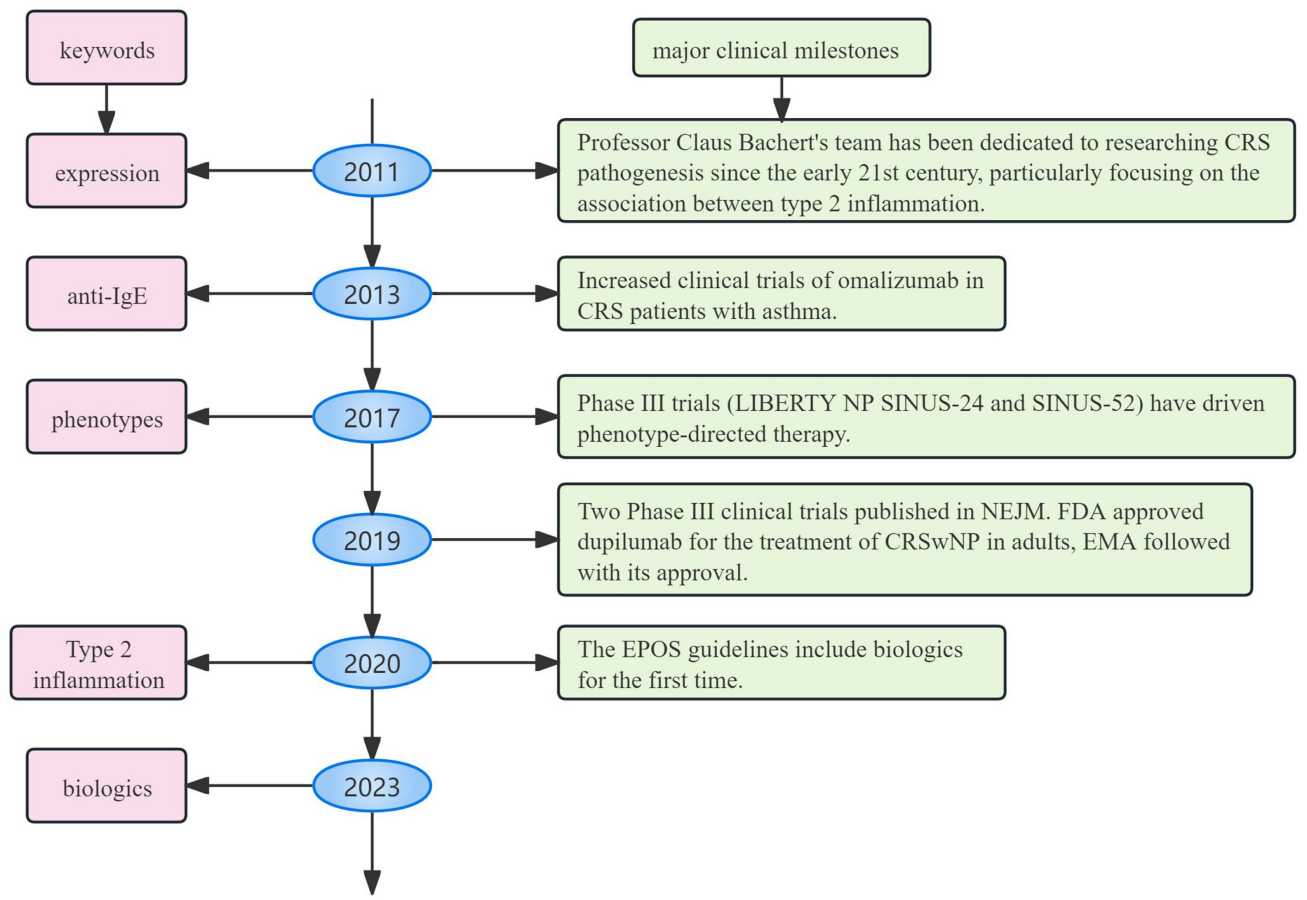
Timeline linking major clinical milestones to bibliometric bursts. It shows how basic research drives clinical change, from basic research to targeted therapy and finally to clinical guidelines.

As depicted in Figure 9, bibliometric bursts closely followed clinical milestones. It is supported by government funding and has an advanced clinical research platform, both Ghent University and Ghent University Hospital experienced a citation surge early on, lasting nearly a decade. Ghent University, a prominent European research center, has played a leading role in early clinical trials on biologic treatments for CRS. Professor Claus Bachert, a distinguished scholar in otolaryngology and immunology at Ghent University, leads the field with 37 articles and 7,287 citations. His team has focused on the pathogenesis of CRS since the early 21st century, particularly the relationship between type 2 inflammation (Th2 pathways) (9), eosinophilic infiltration (10), and nasal polyp formation (11). They have spearheaded research on the mechanisms of action of targets like IL-5 (12, 13) and IgE (14, 15) in CRS, directly advancing biologic treatments and laying the theoretical groundwork for their application. Since 2011, the keyword “expression” has exhibited high burst intensity, reflecting early research on the molecular mechanisms of CRS, such as inflammatory factors and immune pathways (16–18). Following 2013, the keyword “anti-IgE” surged alongside increased clinical trials of omalizumab in CRS patients with asthma (19, 20). The co-morbidity mechanism between asthma and CRS has been widely recognized, and the asthma indication of omalizumab provides a therapeutic approach for CRS. It promotes the emergence of the “phenotypes” concept (which requires the selection of targeted drugs based on phenotype). Starting in 2017, the burst of “phenotypes” marked a shift toward understanding CRS heterogeneity (e.g., type 2/non-type 2 inflammation classification (21, 22)), facilitating the development of personalized treatment strategies. The keyword “innate lymphoid cells” has highlighted research into key effector cells of type 2 inflammation, particularly ILC2 in nasal polyps (23, 24). The high intensity of “eosinophilic asthma” (6.31) underscores the growing focus on the comorbid mechanisms between CRS and eosinophilic asthma (25, 26). More recently, the burst of “nasal polyp score (NPS)” reflects an increased need for quantitative tools to assess the efficacy of biologics, such as endoscopic polyp scores (27). Since 2023, the rising intensities of “biologics” and “biological therapy” indicate a shift from exploratory research to established clinical application (28). Key research gaps include the fact that the top 10 contributing countries account for 80% of global publications, with the United States, Italy, Germany, China, and Japan leading the way. This stems from early research by the Bachert team, which established a global research paradigm and led to rapid FDA/EMA approval. This, in turn, resulted in European and American institutions dominating clinical trials, along with continued government funding for Th2 inflammation research. Furthermore, studies of Chinese and Japanese patients revealed a lower rate of eosinophilic infiltration in Asian CRS patients compared to European and American patients, promoting the exploration of “non-Th2 type” biomarkers. The high research productivity of the United States reflects its triad of advantages in integrating regulatory resources, funding, and academic leadership. However, this may marginalize research priorities in underserved regions, such as tropical areas with high rates of fungal sinusitis (or fungal rhinosinusitis). In contrast, African nations contribute less than 1% of global research output, limiting access to innovative therapies in resource-constrained regions. The primary reasons are the limited accessibility of biologics and insufficient research funding. Although China’s basic research output has increased in recent years, it only accounts for 5% of leading international multicenter trials due to delayed reimbursement coverage by medical insurance. Furthermore, while much of the current research focuses on Th2-type CRS, non-Th2-type CRS remains underexplored, leaving many patients without effective biologic options. At the same time, we hope to strengthen the global distribution of clinical trial sites; establish regional CRS registries; and foster cooperation between pharmaceutical companies and governments to reduce biologic costs in low- and middle-income countries (LMICs).

Through systematic analysis of the scientific literature on biologics in CRS, bibliometrics provides essential insights for the precise management of the condition, particularly in areas such as patient stratification, treatment algorithms, and outcome optimization. Keyword analysis was used to identify dominant biomarkers, and phenotypic classification was defined to pinpoint the population most responsive to biologic agents. Recent trends indicate a shift toward early intervention and combination therapies for moderate and severe CRS patients, aiming to optimize treatment pathways and minimize delays in stepwise management. Additionally, core evaluation indicators were established through high-frequency cited literature, leading to the promotion of a unified efficacy evaluation framework to support individualized treatment goals.

Eosinophilic CRSwNP (eCRSwNP) is a prominent pathological subtype of CRS, characterized by chronic inflammation of the nasal and sinus mucosa, eosinophilic infiltration, and polyp formation. This condition predominantly involves a type 2 inflammatory response with CD8 + T lymphocytes and various immune cells. Traditional treatments show limited efficacy, with high recurrence rates. Biologic agents target critical molecular pathways in type 2 inflammation, directly intervening in the disease’s pathophysiology, rather than merely managing symptoms. Dupilumab blocks IL-4/IL-13 signaling, inhibiting the Th2 inflammatory cascade (29); omalizumab binds free IgE, reducing mast cell and basophil activation (30); mepolizumab inhibits IL-5, reducing eosinophil production and infiltration (31). Dupilumab significantly improves both objective and patient-reported outcomes in CRSwNP, such as loss of smell, and reduces systemic and nasal biomarker levels compared to placebo at week 24 (32). No significant differences were observed between patients with and without allergic rhinitis (AR). Mepolizumab reduces the need for OCS (33), while omalizumab can lower the NPS (34). For patients with concurrent asthma, both nasal symptoms and lung function improve concurrently. Biologic therapy also helps reduce the need for repeated surgeries and lowers the risk of postoperative recurrence.

While biologics have demonstrated efficacy in patients with type 2 inflammation, their clinical application is not without limitations. As a relatively new treatment option, the long-term efficacy and safety of biologics require further validation. Although biologics are currently recommended as second-line treatments in clinical guidelines, some researchers advocate for their early use in high-risk patients to minimize surgical trauma. Long-term data on efficacy and safety are essential to guide the selection of biologics for patients with various phenotypes, ultimately strengthening their position in clinical guidelines based on available evidence. Furthermore, keyword analysis indicates that most current research on biologic agents targets type 2 inflammatory factors, with opportunities remaining for the development of new targets and therapies for non-type 2 sinusitis patients. Research on biologic treatments for CRS in children remains limited, highlighting another gap in the literature. In the future, research resources should prioritize identified gaps in the field, such as developing biologics for non-type 2 sinusitis, large-scale randomized controlled trials (RCTs) and long-term real-world evidence (RWE) on emerging targets in CRS, safety and efficacy evaluations of biologics in specific populations, ultra-long-term (>5–10 years) monitoring of safety, efficacy maintenance, and drug resistance, as well as systematic surveillance and mechanistic studies of rare but severe adverse events. While paying close attention to hot areas, we can promote the in-depth development of research. There can also promote methodological innovation and interdisciplinary collaboration to accelerate knowledge discovery and translational applications using artificial intelligence/machine learning and advanced clinical trial design.

## 5 Limitations

While this study offers valuable insights into research trends and future directions of biologic treatments for CRS, several limitations must be acknowledged. First, the data for this bibliometric analysis were exclusively sourced from the WoS, potentially limiting the completeness of the global research landscape on biologic therapies for CRS. However, the WoS adheres to stringent selection criteria, including journal influence, peer review quality, and publication norms, ensuring the academic authority and reliability of the collected literature. As one of the earliest comprehensive citation databases, it offers extensive historical coverage, making it crucial for trend analysis and long-term impact assessment, which many emerging databases fail to match. Additionally, bibliometric analysis is limited to evaluating the quantity and relevance of publications, rather than their quality. While it provides objective, data-driven insights, the quality of research—encompassing rigorous study design, result reliability, and clinical relevance—remains subjective and cannot be captured by algorithms. For instance, low-quality studies may be frequently cited due to novel topics, or literature later disproven by subsequent research may still appear in co-citation networks. Furthermore, the time lag between the publication and citation of research results means that bibliometric analysis, reliant on historical citation data, may underestimate recent breakthroughs.

## Data Availability

The original contributions presented in the study are included in the article/Supplementary material, further inquiries can be directed to the corresponding author.
